# Excess Mortality in an Inception Cohort of Childhood Diabetes Diagnosed 1990–2010

**DOI:** 10.1155/2024/1844752

**Published:** 2024-03-29

**Authors:** Samantha J. Lain, Lindsay Stevens, Maria E. Craig, Alicia J. Jenkins, Kirstine J. Bell, Alison Pryke, Kim C. Donaghue, Natasha Nassar

**Affiliations:** ^1^Children's Hospital at Westmead Clinical School, The University of Sydney, Sydney, Australia; ^2^Institute of Endocrinology and Diabetes, The Children's Hospital at Westmead, Sydney, Australia; ^3^Discipline of Child and Adolescent Health, University of Sydney, Sydney, Australia; ^4^Discipline of Paediatrics and Child Health, School of Clinical Medicine, UNSW Health, University of New South Wales, Sydney, Australia; ^5^Charles Perkins Centre, University of Sydney, Sydney, Australia; ^6^Baker Heart and Diabetes Institute, Melbourne, Australia

## Abstract

**Objective:**

Evaluate the mortality risk of childhood-onset type 1 diabetes compared to the general population. *Research Design and Methods*. The study population, identified from the Australasian Paediatric Endocrinology Group diabetes register, was diagnosed with type 1 diabetes at age < 16 in New South Wales (NSW), Australia, from 1990 to 2010. The register was linked to National Death Index registrations to ascertain timing and cause of death up to 31/12/2022. Risk factors for mortality were assessed using multivariable Cox regression models and observed mortality rate compared to “expected” rates in the Australian general population using indirect-standardized mortality ratios (SMR), overall and by sex and age at diagnosis. Diabetes-related cause of death categories were identified.

**Results:**

Of 5,417 children diagnosed with type 1 diabetes, 157 subsequently died, with all-cause mortality of 1.37/1,000 person years. Increased mortality risk was associated with living in most disadvantaged areas (aHR 1.81 (1.05, 3.11)) but not living in a rural area. Overall SMR was 2.83 (95% CI 2.40, 3.33) with females having higher SMR than males (4.18 vs. 2.19). Most common causes of death recorded were acute diabetes complications (26%), including diabetes ketoacidosis, accident/misadventure (21%), and chronic diabetes complications (15%). Alcohol and/or drug use contributed to 17% of deaths.

**Conclusion:**

Compared to the general population, higher risk of mortality in people with type 1 diabetes was associated with female sex and living in area of socioeconomic disadvantage. Education about minimizing risk-taking behaviors should be communicated to young adults with type 1 diabetes.

## 1. Introduction

It has been well-documented that childhood-onset type 1 diabetes has a higher rate of mortality compared to the general population. A systematic review examining excess mortality in childhood type 1 diabetes reported a large range of standardized mortality ratios (SMR) compared to the general population, from no excess deaths to 8.5 times higher [1]. However, many studies in the review included a small number of deaths, and mortality data that were over 20 years old. Recent data are important as treatment and management of type 1 diabetes is rapidly evolving. Mortality rates for people with type 1 diabetes have decreased over the last two decades by ∼2.1% per year in Australia and up to 5.8% per year in Denmark [2]; however, as Donzeau et al. [3] identified, few studies of childhood-onset type 1 diabetes have included recent mortality data from 2010 onwards.

Type 1 diabetes mortality rates differ significantly between countries, with an inverse association between mortality and a country's expenditure on health [1]. Australia has one of the lowest all-cause mortality rates for type 1 diabetes [2], and deaths in younger people are rare. Studies from two Australian states, Victoria and Western Australia, have reported SMRs for young adults (aged < 40 years) with childhood-onset type 1 diabetes compared to the general population of 1.9 (95% CI, 0.7–4.3) [4] and 3.3 (95% CI 2.0–5.2) [5], respectively; however, both studies included a small number (≤20) of mortality events. Studies with large sample sizes are important for precise estimates of SMRs.

Sex differences between SMRs for people with type 1 diabetes have been well-documented, with higher SMRs consistently reported for females [6]. Another factor that may be important to investigate in relation to mortality rates of people with type 1 diabetes is age at diagnosis; however, this factor has been less frequently examined and studies have shown conflicting results [7–9]. Recent studies have identified endotypes of type 1 diabetes, whereby those diagnosed in early childhood (<7 years) have a more aggressive disease [10]. To our knowledge, the association between diagnosis of type 1 diabetes in young children (≤7 years) and mortality has not been examined but may be higher given the increased window of time that they are vulnerable to hypoglycemia. This study aims to utilize contemporary data to investigate the main causes of death for people with childhood-onset type 1 diabetes, their rate of mortality relative to the general population, and examine the impact of various factors, including sex, age of diagnosis, and socioeconomic status on mortality risk.

## 2. Materials and Methods

### 2.1. Study Setting and Participants

The study population comprised children diagnosed with type 1 diabetes before their 16th birthday in New South Wales (NSW), Australia, from 1990 to 2010. Children were excluded if they had diabetes secondary to another primary condition (including cystic fibrosis, thalassemia major, pancreatectomy for neonatal hyperinsulinism), or if they did not reside in NSW at the time of diagnosis.

### 2.2. Type 1 Diabetes and Death Data

The NSW Australasian Paediatric Endocrinology Group (APEG) diabetes register has prospectively collected data about children with type 1 diabetes in NSW since 1990. Newly diagnosed children with type 1 diabetes are reported to the APEG register by pediatricians, physicians, pediatric endocrinologists, diabetes educators, and nurses. The criteria to be included in the APEG register are (1) diagnosis of type 1 diabetes by a physician, (2) diagnosis defined by date of commencement of insulin therapy before the 16th birthday, and (3) place of usual residence at diagnosis within NSW [11].

The APEG register was linked to the National Death Index (NDI) to identify deaths. The NDI includes registration of all deaths in Australia and data on date of death and age of death. The NDI codes underlying and, up to 20, contributing causes of death according to the 10th revision of the International Classification of Diseases (ICD10). The NDI is available from 1990 to 2022. Those who died in 2022 are identified; however, cause of death information was not available as time is needed for coding and cleaning. Data linkage between APEG and NDI were performed by the Australian Institute of Health and Welfare (AIHW).

### 2.3. Explanatory Variables

Child sex, age at diagnosis, year of diagnosis, and Statistical Local Areas (SLA) were obtained from the APEG register. Age at diagnosis was dichotomized to those diagnosed before age 8 years (younger childhood onset) and those diagnosed from 8 to 15 years of age (older childhood onset). SLA identifies the area of residence at time of diagnosis and was used to categorize socioeconomic status (SES) and remoteness of residence. The Socio-Economic Index for Areas was used to categorize SLA in SES quintiles from most disadvantaged (Q1) to most advantaged (Q5) [12]. The remoteness of residence was classified using the Accessibility/Remoteness Index of Australia (ARIA) and categorized into two groups (major cities and regional or remote) [13].

### 2.4. Statistical Analysis

Characteristics of the study cohort were explored using descriptive statistics and differences between those who died and those who did not were evaluated using *χ*^2^ tests. To examine the association between explanatory variables and mortality, multivariable Cox regression models were used to account for different follow-up periods of children in the cohort and adjusted hazard ratios (aHRs) with 95% CIs reported. Individuals were followed until the earliest of their date of death or study end (December 31, 2022). To examine the observed mortality rate in the type 1 diabetes cohort compared to the general population in Australia, indirect SMRs were calculated. The person-years at risk were calculated for each child in the APEG cohort, and a crude mortality rate was calculated by sex and age at death (10-year age groups, 10–19, 20–29, and 30–39 years). The expected number of deaths for each sex and age group was identified from the annual population statistics and mortality data for NSW published by the Australian Bureau of Statistics [14] and standardized to population rates in 2015 [15], the median year of death for the APEG cohort. SMRs, defined as the ratio of total observed to total expected deaths per 100,000 person-years, were calculated for sex and younger/older age of diagnosis, and 10-year attained age groups. The 95% confidence intervals (CIs) for SMRs were derived from methods detailed by Rothman [16] and Greenland for SMRs. All analyses were performed using SAS, version 9.4 software (SAS Institute).

The causes of death were reviewed in consultation with pediatric endocrinologists (KD, MC) and grouped according to those attributable to a diabetes-related condition. These included, in order of relevance, (i) acute diabetes-related complication (e.g., diabetic ketoacidosis (DKA) or hyperosmolarity, hyperglycaemia, cerebral oedema); (ii) chronic diabetes-related complication (e.g., chronic kidney condition, complication related to amputation); (iii) diabetes mellitus without complication; (iv) cancer; (v) cardiovascular disease not associated with diabetes; (vi) accident/misadventure; and (vii) other—all remaining cases (Supplementary File S1). Given each person could have up to 20 causes of death recorded, one diabetes-related cause of death was assigned according to the seven groups using a hierarchical approach. The frequency and proportion of individuals in each group were then compared overall, and by sex, age at death, and age at diagnosis.

The contribution of drug and alcohol use to all deaths was examined separately by reviewing all cause of death fields (see Supplementary File S1 for codes).

### 2.5. Data and Resource Availability

The data that support the findings of this study are available from the Australian Institute of Health and Welfare, but restrictions apply to data availability, which were used under license for the current study and therefore are not publicly available.

## 3. Results

Of 5,417 children under the age of 16 years diagnosed with type 1 diabetes in NSW from 1990 to 2010, 157 died (2.9%). Median age at follow-up for the cohort was 30 years of age. This differed by age of diagnosis; median age at follow-up of 26 years for those diagnosed before age 8, and 33 years for those diagnosed with type 1 diabetes aged 8–15 years. Cause of death data was available for 149 (95%) deaths, and 18 of these deaths (12%) occurred outside NSW and were recorded in a different Australian state.

### 3.1. All-Cause Mortality

The crude all-cause mortality rate was 137 per 100,000 person-years. The majority (73%) of deaths were recorded from 2010 to 2022. Males had an all-cause mortality rate of 143 per 100,000 person-years compared to 131 per 100,000 person-years for females. There were higher rates of mortality amongst youth who were diagnosed at a later age (8–15 years), those who lived in lowest SES quintile, and those who were diagnosed in the decade 1990–1999 (Table 1). After adjustment for the explanatory variables and follow-up time, children who were diagnosed after age 8 years had an increased risk of death (aHR 1.87, 95% CI 1.31, 2.67) compared to those diagnosed before aged 8 years, and children living in most disadvantage areas (lowest SES quintile) had an increased risk of death (aHR 1.81 95% CI 1.05, 3.11) compared to those who lived in the least disadvantaged areas (highest SES quintile, Table 1). There was not a significant difference in risk of death between those that resided in metropolitan areas compared to those residing in rural or remote areas.

### 3.2. Standardized Mortality Ratios

When comparing mortality rates of those with type 1 diabetes to the general population, the APEG cohort had higher overall mortality (SMR 2.83 95% CI 2.40, 3.33, Table 2). The SMRs increased with attained age, and females had a higher SMR of 4.18 (95% CI 3.30, 5.30) compared to males, 2.19 (95% CI 1.75, 2.74, Table 2). While the crude mortality rate between females and males with type 1 diabetes was similar, the difference in rates appears to be influenced by the much lower expected mortality rate for females than for males in the general population (Table 2). The group with the highest SMR were females aged 30–39 years, who were five times more likely to die compared to a female of this age in the general population (SMR 5.06, 95% CI 3.30, 7.76).

People who were diagnosed with type 1 diabetes before the age of 8 years had an overall SMR of 1.94 (95% CI 1.39, 2.72) and those diagnosed with type 1 diabetes from the age of 8–15 years had an overall SMR of 3.29 (95% CI 2.73, 3.97) (Table 3). The crude mortality rates and SMR for deaths before age 30 years did not differ between those with younger childhood-onset compared to those with older childhood-onset type 1 diabetes. The group that had the highest SMR were those diagnosed with type 1 diabetes aged 8–15 years and died aged 30–39 years (4.33 95% CI 3.24, 5.78); however, due to small numbers the SMR for those diagnosed before age 8 and died aged 30–39 years was imprecise with wide confidence intervals (Table 3).

### 3.3. Diabetes-Related Cause of Death

**Figure 1:**
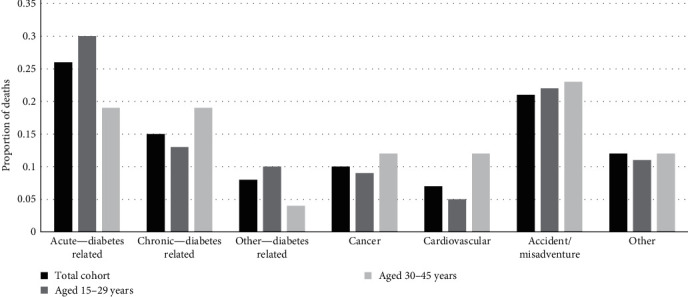
Diabetes-related cause of death stratified by age at death.

Just over a quarter (26%) of those who died had a diabetes-related cause of death recorded as acute complications from type 1 diabetes (Figure 1). Of these, 59% had a record of diabetic with ketoacidosis, 15% record of diabetic with coma, and 23% had a record of hypoglycemia. Other causes of death included; accident and misadventure (21%), chronic complications of type 1 diabetes (15%), cancer (10%), and cardiovascular conditions, that were not recorded as a complication of type 1 diabetes (7%) (Figure 1). For 19% (29/149) of deaths, a cause of death of “diabetes without complication” (ICD10 code E10.9, E14.9) was recorded; however, all but 12 (8%) could be categorized into acute or chronic diabetes-related complication using contributing cause of death codes. Seventeen percent (25/149) of deaths had a cause of death that was drug or alcohol-related, predominantly for those aged 20–35 years. Amongst this group, 9% (13/149) had an underlying cause of death recorded as “accidental or intentional poisoning.” Most cases recorded drug use, including alcohol, cannabis and opioids, as a contributing cause of death.

When comparing diabetes-related causes of death across age groups, a greater proportion of people aged 30–45 years died of chronic complications, cardiovascular causes, and cancer compared to those who died at younger ages. While a higher proportion of those aged <30 years died of acute complications (Figure 1). A greater proportion of males died from accident and misadventure compared to females (18% vs. 6%, *p*=0.05).

## 4. Discussion

In this study of mortality in a cohort of Australians with type 1 diabetes diagnosed in childhood, we found that people with type 1 diabetes had an overall SMR of 2.83 (95% CI 2.40, 3.33) compared to the general population. While women with type 1 diabetes had similar crude mortality rate to men with type 1 diabetes, they had SMRs four times that of the general female population. Those who reside in areas of socioeconomic disadvantage had a higher risk of mortality amongst the cohort; however, those residing in rural or remote areas of NSW did not differ from those living in metropolitan areas.

The overall SMR of 2.83 for childhood-onset type 1 diabetes in this study compares with recent Australian [4, 5] and European studies reporting an increased risk of mortality in adolescence and young adulthood 2–3 fold that in the general population [3, 17, 18]. Interestingly, we have included recent mortality data up to 2022; however, relative mortality rates for people with type 1 diabetes have not decreased. Characteristics that we found had significantly higher mortality rates relative to the general population, age attained [19], and female [6], were also consistent with the literature. Children that resided in an area of high socioeconomic disadvantage with type 1 diabetes had over double the risk of mortality compared to those who lived in areas that were least disadvantaged, also previously reported [5, 20, 21].

We found that SMRs increased with age attained, with those in the cohort who reached 30–39 years having four times the risk of mortality of the general population. This is consistent with SMRs reported for this age group in an Australian study of all people with type 1 diabetes, including those diagnosed in adulthood [19]. That study, and others, found that mortality relative to the general population for those with type 1 diabetes began to decrease after the age of 50 years back to around double the risk of the general population for those aged in their 60s and 70s [22, 23]. Longer term data are required to examine whether this is true for those diagnosed with type 1 diabetes in childhood in Australia.

Contrary to expectation, children diagnosed with type 1 diabetes at a young age (<8 years) were found to have a lower risk of mortality in adolescence and young adulthood compared to those diagnosed in later childhood (aged 8–15 years) when adjusted for other characteristics. However, this finding is an artifact of the data collection with lower median age at follow-up for those with younger childhood-onset type 1 diabetes and increasing mortality rates over age 30 years. When crude mortality rates are examined in SMR calculation, prior to age 30 years, there was no difference in mortality by age of diagnosis. Only a small number of people with type 1 diabetes diagnosed before age 8 reached 30–39 years, and few (<5) died at this age, giving imprecise SMR estimates and wide confidence intervals. This analysis needs to be repeated when additional follow-up is available. This is consistent with a type 1 diabetes registry study in Norway that showed cumulative mortality was significantly higher in those diagnosed with type 1 diabetes earlier (<10 years); however, SMRs were not significantly different between groups [17].

The main diabetes-related cause of death for our cohort was due to acute complications from type 1 diabetes (26%). Most of these deaths were recorded as having ketoacidosis; however, for those with a diagnosis code related to “diabetes with coma,” the ICD10 coding does not differentiate between hypoglyemic coma and hyperglycemic coma (DKA) [24]. For people aged less than 30 years when they died, more died from acute complications from diabetes than any other cause. Similar findings have been reported in Norway and Sweden [24, 25]. Although over the last 20 years, there have been a number of advances in treatment and improved education for people with diabetes aimed at achieving glycemic targets and reducing diabetic complications, including availability of continuous glucose monitoring and insulin pump therapy [26], Australian hospital admission rates for adolescents with DKA have not changed [27].

Almost a fifth (19%) of our cohort had a cause of death recorded as “diabetes mellitus without complications.” While there was additional cause of death information to further categorize some deaths, for 8% the cause of death was unclear. A similar proportion (19%) of deaths amongst young adults with childhood-onset type 1 diabetes had this recorded cause of death in Norway, and after closer review of additional documentation almost half were categorized as “sudden unexplained death” [25]. Sudden unexpected death includes death where no potentially fatal or critical event or disease was present at least 24 hr before death [28] and can occur up to 10 times more often in young people with type 1 diabetes compared to the general population [29]. Although there is no known cause of these deaths, some suggest these deaths may be attributable to severe hypoglycemia, or arrythmia potentially induced by hypoglycemia itself and related electrolyte disturbance [29]. Future research into predictors of sudden unexpected death among young people with type 1 diabetes is needed.

For people aged 20–39 years, accident and misadventure caused a quarter of deaths, with accidental or intentional poisoning accounting for half of these. In this study, 13 cases per ∼100,000 person-years had an underlying cause of death recorded as accidental or intentional poisoning, double the annual rate reported in the general population of Australia (6–8 per 100,000 population died every year from drug-induced death) [30]. Overall, 17% of deaths have alcohol or drug use recorded. The rates of alcohol and drug use in young adults with type 1 diabetes are reported to be similar to the general population; however, alcohol and drug consumption can impact blood glucose levels causing hypoglycemia and hyperglycemia [31]. The physiological impacts of drugs and alcohol specific to people with type 1 diabetes should be communicated by clinicians, particularly during transition from pediatric to adult care, and harm reduction techniques conveyed [32].

The main strength of this study is the large, population-based inception cohort of childhood-onset type 1 diabetes that has been linked to up-to-date national mortality data. The inclusion of mortality data from states outside NSW identified an additional 18 deaths. The contemporary dataset incorporates people with type 1 diabetes managed using the most up-to-date clinical guidelines [33]. The main limitation of this study was the lack of clinical information on glycemia, diabetes management, and access to services to determine their impact on mortality. Pregnancy status of women with type 1 diabetes was also not available. Although we had a 30-year follow-up for some of the cohort, those diagnosed after 2000 had a shorter follow-up time and their outcomes should be examined in future research.

## 5. Conclusions

In conclusion, compared to the general population, young adults with childhood-onset type 1 diabetes had higher risk of mortality, and this increased risk has not changed with recent improvements in treatments. Ongoing communication of harm reduction techniques, such as minimizing risk-taking behavior with drugs and alcohol, and targeted education about glycemic control, particularly following transition from pediatric to adult care, is required.

## Figures and Tables

**Table 1:** Characteristics of type 1 diabetes cohort and adjusted risk of mortality.

Chracteristics	Total cohort	Survived, *N* (%)	Died, *N* (%)	Chi-sq. *p*	aHR^*∗*^ (95% CI)
	5,417	5,260 (97)	157 (3)	—	—
Sex	—	—	—	0.43	—
Male	2,761 (51)	2,676 (51)	85 (54)	—	1.20 (0.86, 1.62)
Female	2,651 (49)	2,579 (49)	72 (46)	—	—
Age at diagnosis	—	—	—	<0.01	—
0–7	2,126 (39)	2,085 (40)	41 (26)	—	—
8–15	3,291 (61)	3,175 (60)	116 (74)	—	1.87 (1.31, 2.67)
Year of diagnosis	—	—	—	<0.01	—
1990–1999	2,294 (42)	2,185 (42)	109 (69)	—	1.54 (1.06, 2.22)
2000–2009	3,123 (58)	3,075 (58)	48 (31)	—	—
Socioeconomic status	—	—	—	0.07	—
Most disadvantaged	955 (18)	920 (18)	35 (22)	—	1.81 (1.05, 3.11)
Quintile 2–4	3,352 (62)	3,252 (62)	100 (64)	—	1.42 (0.88, 2.30)
Least disadvantaged	1,102 (20)	1,080 (21)	22 (14)	—	—
Area of residence	—	—	—	0.15	—
Major cities	3,665 (68)	3,567 (68)	98 (62)	—	—
Regional or remote	1,744 (32)	1,685 (32)	59 (38)	—	1.13 (0.81, 1.58)

^*∗*^Hazard ratios adjusted for all characteristics in table.

**Table 2:** Standardized mortality ratios by age attained and sex for childhood-onset type 1 diabetes compared to the general population.

Attained age (years)	Sex	Person-years	Crude mortality rate per 100,000	Expected mortality rate per 100,000 (2015 Aus)	SMR (95% CI) indirect
10–19	Female	22,941	56.67	16.53	3.43 (1.99, 5.91)
10–19	Male	22,923	56.71	26.47	2.14 (1.24, 3.69)
10–19	Total	45,864	56.69	21.63	2.62 (1.78, 3.85)
20–29	Female	20,986	162.01	24.15	6.71 (4.79, 9.39)
20–29	Male	21,666	161.54	63.81	2.53 (1.82, 3.52)
20–29	Total	42,653	161.77	44.22	3.66 (2.89, 4.63)
30–39	Female	8,156	257.49	50.93	5.06 (3.30, 7.76)
30–39	Male	8,537	328.00	101.25	3.24 (2.24, 4.69)
30–39	Total	16,692	293.55	76.05	3.86 (2.92, 5.11)
Total	Female	52,083	130.56	52.83	4.18 (3.30, 5.30)
Total	Male	53,126	143.06	98.33	2.19 (1.75, 2.74)
Total	Total	105,209	136.87	75.70	2.83 (2.40, 3.33)

**Table 3:** Standardized mortality ratios by age attained and early (<8 years) and late (8–15 years) age at diagnosis for childhood-onset type 1 diabetes compared to the general population.

Attained age (years)	Age at diagnosis	Person-years	Crude rate per 100,000	Expected mortality rate per 100,000 (2015 Aus)	SMR (95% CI) indirect
10–19	Early	20,685	48.34	21.63	2.24 (1.21, 4.16)
10–19	Late	25,179	63.55	21.63	2.94 (1.80, 4.80)
10–19	Total	45,864	56.69	21.63	2.62 (1.78, 3.85)
20–29	Early	12,695	165.42	44.22	3.74 (2.44, 5.74)
20–29	Late	29,957	160.23	44.22	3.62 (2.73, 4.80)
20–29	Total	42,653	161.77	44.22	3.66 (2.89, 4.63)
30–39	Early	2,731	109.84	76.05	1.44 (0.46, 4.46)
30–39	Late	13,961	329.49	76.05	4.33 (3.24, 5.78)
30–39	Total	16,692	293.55	76.05	3.86 (2.92, 3.97)
Total	Early	36,111	94.15	75.70	1.94 (1.39, 2.72)
Total	Late	69,097	159.20	75.70	3.29 (2.73, 3.97)
Total	Total	105,209	136.87	75.70	2.83 (2.40, 3.33)

## Data Availability

The data that support the findings of this study are available from the Australian Institute of Health and Welfare, but restrictions apply to data availability, which were used under license for the current study and therefore are not publicly available.

## Supplementary Materials

Supplementary Materials Tables of cause of death categories and associated ICD10 coding.
