# Supplementary material for: Excess Mortality in an Inception Cohort of Childhood Diabetes Diagnosed 1990–2010
Source: Pediatr Diabetes. 2024 Mar 29;2024:1844752. doi: 10.1155/2024/1844752 (PMC12016878; doi:10.1155/2024/1844752)
Supplement: Supplementary Materials — Tables of cause of death categories and associated ICD10 coding. [file 1844752.f1.docx]

**Supplementary file 1: Cause of death categories and associated ICD10 coding**

Cause of death ICD10 codes included in ‘Acute diabetes-related complications’

| ICD10-AM code | ICD10 description |
| --- | --- |
| E10.0 | Type 1 diabetes mellitus with hyperosmolarity |
| E10.1 | Type 1 diabetes mellitus with acidosis |
| E14.0 | Unspecified diabetes mellitus with hyperosmolarity |
| E14.1 | Unspecified diabetes mellitus with acidosis |
| E16.2 | Hypoglycaemia, unspecified |
| E27.2 | Addisonian crisis |
| G93.1 | Anoxic brain damage, not elsewhere classified |
| G93.6 | Cerebral oedema |
| J45.9 | Asthma |
| N17.9 | Acute kidney failure, unspecified |
| R55 | Syncope and collapse |
| R73.9 | Hyperglycaemia, unspecified |
|  |  |

Cause of death ICD10 codes included in ‘Chronic diabetes-related complications’

| ICD10 code | ICD10 description |
| --- | --- |
| E10.2 | Type 1 diabetes mellitus with kidney complication |
| E10.7 | Type 1 diabetes mellitus with multiple complications |
| E10.8 | Type 1 diabetes mellitus with unspecified complication |
| E14.2 | Unspecified diabetes mellitus with kidney complication |
| E84.0 | Cystic fibrosis with pulmonary manifestations |
| E84.8 | Cystic fibrosis with other manifestations |
| E84.9 | Cystic fibrosis, unspecified |
| E87.5 | Hyperkalaemia |
| N18.5 | Chronic kidney disease, stage 5 |
| N18.9 | Chronic kidney disease, unspecified |
| N28.9 | Disorder of kidney and ureter, unspecified |
|  |  |
| Y83.0 | Surgical operation with transplant of partial or whole organ |
| Y83.2 | Surgical operation with anastomosis, bypass or graft |
| Y83.5 | Amputation of limb(s) |

Cause of death and ICD codes included in other respective categories

| ICD10 code | ICD10 description |
| --- | --- |
| E10.9, E14.9 | Diabetes mellitus without complication |
| C00-D49 | Cancer |
| I00-I99 | Cardiovascular disease, not associated with diabetes |
| V00-Y98 | Accident and misadventure (including intentional injuries) |

Cause of death ICD10 codes that included alcohol and drug use

| ICD10 code | ICD10 description |
| --- | --- |
| X40-X49 | Accidental poisoning by and exposure to noxious chemical |
| X60-X69 | Intentional self-poisoning by and exposure to noxious chemical |
| F10-F17, F18, F19 | Mental and behavioural disorders due to use of alcohol and drugs |
| T36-T51 | Poisoning by drugs, medicants and alcohol |
